# Surface enhanced Raman scattering for the multiplexed detection of pathogenic microorganisms: towards point-of-use applications

**DOI:** 10.1039/d1an00865j

**Published:** 2021-08-31

**Authors:** Matthew E. Berry, Hayleigh Kearns, Duncan Graham, Karen Faulds

**Affiliations:** Centre for Molecular Nanometrology, Department of Pure and Applied Chemistry, Technology and Innovation Centre, University of Strathclyde 99 George Street Glasgow G1 1RD UK karen.faulds@strath.ac.uk

## Abstract

Surface enhanced Raman scattering (SERS) is a technique that demonstrates a number of advantages for the rapid, specific and sensitive detection of pathogenic microorganisms. In this review, an overview of label-free and label-based SERS approaches, including microfluidics, nucleic acid detection and immunoassays, for the multiplexed detection of pathogenic bacteria and viruses from the last decade will be discussed, as well as their transition into promising point-of-use detection technologies in industrial and medical settings.

## Abstract 2

In this review we discuss surface enhanced Raman scattering (SERS) based techniques for the multiplexed detection of pathogenic microorganisms and their transition into point-of-use detection technologies in the field.

## Graphical abstract

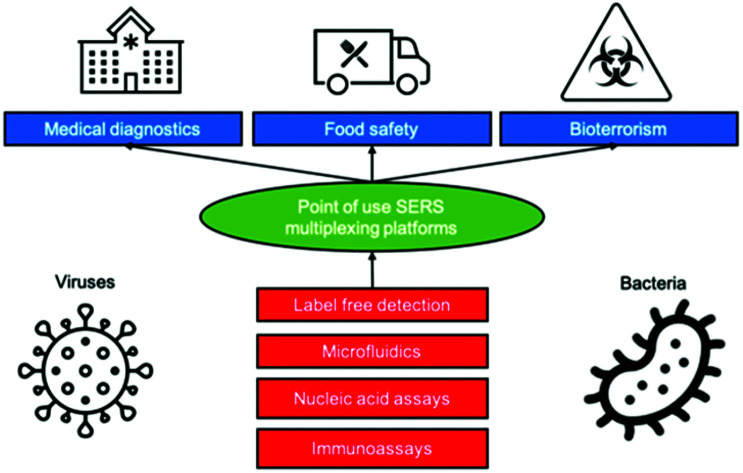

## 1. Current challenges in the detection of pathogenic microorganisms

Pathogenic microorganisms are capable of causing a vast array of different disease states within animal and human communities. This term includes bacterial and viral strains that can be transferred between hosts by a number of different transmission routes including food, water, bodily fluids and air. When infectious diseases become prevalent in a large enough number of people, public health and socioeconomic crises can rapidly ensue.^1^ Strategies for the successful detection of pathogens in a rapid and accurate manner that can be used at the point of need are critical in preventing such incidences and ensuring the safety and health of the general public.^2^ The introduction of sufficiently fast and precise methodologies would prevent the release of contaminated food products to the public, allow clinicians to identify pathogens in patients before they develop life-threatening complications from conditions such as septicaemia, meningitis and pneumonia, and better inform governments of the state on emerging and endemic infectious disease events such as the ongoing global pandemic caused by the severe acute respiratory syndrome coronavirus 2 (SARS-CoV-2).^3–9^

The most common techniques for the detection of pathogenic microorganisms are based on microbiological, morphological and biochemical identification processes that are time consuming and often laborious.^10^ Diagnostic tools such as cell culture, polymerase chain reaction (PCR) and immunology-based techniques including enzyme-linked immunosorbent assays (ELISA) have been applied to accurately detect both bacterial and viral strains in many different settings.^11–15^ These methods have become regarded as the ‘gold standard’ of pathogen identification because they can provide sensitive, qualitative and quantitative information on microorganisms, however, they all have associated disadvantages. For example, cell culture methods require incubation and enrichment steps requiring up to 24 hours in selective media often followed by an additional 1–3 days for confirmation by biochemical tests.^16^ Furthermore, the reliability of culture is linked to the composition of the enrichment broth and the vitality of the organism under investigation, and these methods require specialised and expensive operational equipment such as incubators, autoclaves and microbiological safety cabinets. PCR and ELISA can achieve much faster identification in a time range of several hours, but they are subject to enzyme efficiency, which has proven problematic because these techniques are susceptible to contamination.^17^ Operationally, they require skilled personnel, prior knowledge of the strains under investigation, expensive equipment, and sometimes additional culturing to enrich samples, thereby limiting their application as point-of-use (POU) testing platforms in the field. A testing strategy is considered POU if it provides rapid ‘on-site’ results at the site of care delivery, production, or in resource limited settings, supporting timely and proper preventative action.^18^ These inherent constraints are being circumvented by the rise of biosensor technology because it has the potential to provide sensitive, specific and reliable data comparable to conventional techniques, but with shorter analysis times and less restrictions on field deployability.^19^ Whilst there are a number of POU biochemical tests available for the detection of pathogenic organisms, such as adenosine triphosphate (ATP) bioluminescence and colorimetric lateral flow assays (LFA), they currently suffer from technical challenges pertaining to a lack of specificity and sensitivity compared to the more traditional detection techniques.^20,21^

One emerging biosensor technology is surface enhanced Raman scattering (SERS). SERS is a sensitive and selective analytical technique that involves the detection of target molecules attached to or in very close proximity to the surface of noble metal nanoparticles (NPs) such as gold and silver.^22^ SERS relies primarily on the strength and consistency of plasmonic enhancements caused by the oscillation of free conduction electrons on the metal NP surface. The localised electric fields produced by this oscillation allow for large increases in signal intensity for target molecules orders of magnitude greater than those typically reported for conventional Raman scattering. However, the enhancements can vary largely depending on the laser, substrate and analyte used.^23^ Enhancements of up to 10^14^ are possible when the laser frequency is tuned with an electronic transition within an analyte; this phenomenon is known as surface enhanced resonance Raman scattering (SERRS).^24^ The main advantage of Raman based techniques over other common optical techniques such as fluorescence, which is regularly used in combination with PCR and ELISA, is the sharp, molecularly specific fingerprint spectra obtained that make it possible to discriminate between individual components in a complex sample mixture, allowing for the simultaneous detection of multiple analytes.^25–27^ Other advantages of Raman based techniques include the vast library of Raman active compounds available for preparing SERS nanotags with unique fingerprint spectra, the reduced susceptibility of SERS nanotags to photobleaching when compared with commonly used fluorophores, the compatibility of Raman spectroscopy with aqueous samples which are common in biological applications as well as reduced sample volume requirements and short assay times.^28,29^ The numerous advantages presented here have garnered significant attention throughout the scientific community, with vast improvements being reported in the design, fabrication and synthesis of reproducible and reliable nanostructures for SERS, and it is because of this that SERS-based approaches have been realised for biomedical analysis including the quantitative detection of biomarkers, nucleic acids and cells associated with cancers and infectious diseases.^30–36^

In this review, a chronological overview of label-free and label-based SERS approaches, including microfluidics, nucleic acid detection and immunoassays, for the multiplexed detection of pathogenic organisms from the last decade will be discussed, as well as the recent advancements made in transitioning SERS approaches into promising POU applications and the future steps needed to make SERS a reliable technique in field settings such as food production sites, water facilities and medical clinics. A summary of the approaches and specific applications included within this review is provided for the reader in Table 1.

**Table 1:** A summary of the general approaches and studies discussed within this review

SERS approach	Summary	Performance
(1) Label free detection	◁ SERS substrates for the capture of pathogens	◁ Detection of three Gram-negative bacterial strains (LOD 10^5^ CFU mL^−1^) (ref. 46)
	◁ Intrinsic vibrational fingerprint of pathogens detected (no Raman reporters used)	◁ Detection of *E*. *coli* and *S*. *aureus* in tap water and milk (LOD 10^3^ CFU mL^−1^, 10 min) (ref. 47)
		◁ Detection of multiple bacterial strains (LOD 1 CFU mL^−1^, 5 min) (ref. 48)
		◁ Detection of bacterial strains in mung bean sprouts (LOD 10^2^ CFU mL^−1^, 4 hours) (ref. 49)
		◁ Sampling and detection of bacterial pathogens from skin wound (LOD 10^6^ CFU mL^−1^, 8 hours (5 min for sampling)) (ref. 95)
		◁ Detection of meningitis pathogens in clinical CSF (ref. 99)
(2) Microfluidics	◁ Sample preparation, reaction, separation, and detection integrated into a single device	◁ Label-free detection of *E*. *coli* and *S*. *aureus* in blood (LOD 10^5^ CFU mL^−1^ 100 CFU mL^−1^ in culture) (ref. 53)
	◁ Supports label-free and label-based detection	◁ Label-free detection of three bacterial strains in serum (LOD 4 CFU mL^−1^, 15 min) (ref. 55)
		◁ Label-based detection of *S*. *enterica* and *N*. *lactamica* using DEP enrichment (LOD 70 CFU mL^−1^, 10 min) (ref. 57)
		◁ Label-based detection of three bacterial pathogens at millilitre scale in blood (LOD < 100 CFU mL^−1^, 13 min) (ref. 59)
		◁ Label-based detection of *E*. *coli* using DEP enrichment (LOD 1 CFU mL^−1^) (ref. 61)
		◁ Detection of eight foodborne pathogens (ref. 94)
		◁ Label-free detection of multiple viral strains in clinical nasopharyngeal swabs (ref. 102)
(3) Nucleic acid-based detection	◁ Coupling of assays for the detection of pathogenic DNA with SERS substrates	◁ Detection of meningitis pathogenic DNA (LOD in pM range). (ref. 68) Detection of pathogen DNA in clinical CSF (ref. 69)
	◁ SERS nanotags functionalised with DNA/RNA aptamers	◁ Detection of KSHV and BA DNA using LFA (LOD in fM range) (ref. 70)
	◁ Supports label-based detection	◁ Detection of *S*. *aureus* and *S*. *typhimurium* using aptamer-based magnetic sandwich assay (LOD 15 CFU mL^−1^) (ref. 74)
		◁ Detection of *E*. *coli* and *S*. *aureus* using aptamer-based magnetic sandwich assay in urine samples (LOD 50 CFU mL^−1^, 1.5 hours, 20 CFU mL^−1^ and 15 min for culture) (ref. 75)
		◁ Detection of *E*. *coli* using aptamers in beef samples (LOD 100 CFU mL^−1^, 20 min) (ref. 77)
		◁ Detection of influenzae A H1N1 in complex mixtures (LOD 97 pfu mL^−1^, 20 min) (ref. 78)
		◁ Detection of DNA from 11 common RTI pathogens using LFA (LOD in fM range, 20 min). Detection from throat swab (ref. 106)
		◁ Detection of *S. enterica* and *L*. *monocytogenes* in milk, chicken and beef using LFA and RPA (LOD 22 CFU mL^−1^) (ref. 107)
		◁ Detection of plant pathogens on commercial crops using RPA outside of laboratory (ref. 110)
		◁ Real time detection of MRSA genes using miniaturized PCR system (ref. 111)
(4) Immunoassays	◁ Specific binding of pathogen antigen and antibodies coupled with SERS nanotags	◁ Detection of Zika and dengue biomarkers using dipstick immunoassay (LOD 0.72 ng mL^−1^) (ref. 82)
	◁ Supports label-based detection	◁ Detection of multiple viral strains using magnetic LFA (LOD 10 pfu mL^−1^). (ref. 85) Detection in blood, serum and sputum (LOD 10^5^ pfu mL^−1^)
		◁ Detection of three bacterial pathogens using magnetic sandwich assay (LOD 10 CFU mL^−1^). (ref. 86) Same LODs confirmed on portable system (1 hour) (ref. 104)
		◁ Detection of multiple viral and bacterial pathogens in serum using magnetic sandwich assay (LOD 10 pg mL^−1^) (ref. 87)

## 2. SERS multiplexing techniques for the detection of pathogenic microorganisms

In the context of analytical science, multiplexing refers to the detection of several targets simultaneously in complex sample mixtures. Selective and sensitive multiplexed detection of biochemical targets, such as pathogenic microorganisms or associated biomarkers, is highly relevant in the fields of clinical diagnostics, food safety and bioterrorism because it has the potential to reduce time and cost and allow for more information to be obtained from a single sample.^27^ SERS is highly advantageous for the multiplexed detection of biological targets because it provides the user with the ability to analyse samples with minimal preparation specifically and sensitively.^24^ Furthermore, SERS platforms that are sensitive and generate highly reproducible optical readouts can provide quantitative analysis of a target at clinically relevant concentrations which is often essential for distinguishing between contaminated and uncontaminated samples and allowing the end user to make informed preventative decisions.^34,35^ However, despite the undoubtedly significant improvements in synthetic nanotechnology over the last decade, arguably the greatest challenge in the wider adoption of multiplexing techniques is in the availability of high-quality SERS nanoprobes that are stable in biological systems, specific to biomolecular targets whilst being bioorthogonal and not cross-reactive with other species within sample mixtures.^37,38^ The examples highlighted in the following subsections demonstrate that reliable substrate and assay design, that takes many factors into account including surface attachment, aggregation, and pH, has been realised that a number of diverse strategies can be used for the multiplexed, quantitative detection of pathogenic organisms and associated biomarkers or nucleic acids.

### 2.1. Label-free approaches

Label-free SERS approaches for the detection of microorganisms typically involve the mixing of cells with noble metal NPs in solution or the preparation of SERS substrates for the capture of cells, allowing for the intrinsic vibrational fingerprint of pathogens to be detected and the species or strain of pathogen to be identified.^39–42^ On the most fundamental level, all label-free SERS strategies for the detection of pathogenic microorganisms involve sampling, measurement and spectral analysis stages. In the sampling stage, pathogens are isolated from biological or environmental media before being incorporated into or exposed to a SERS active substrate. After measurements have taken place, the SERS spectra are analysed to filter out the biochemical information pertaining to the target of interest. When multiple pathogens are present, spectral analysis must allow for the discrimination and classification of pathogenic strains based on their distinctive biochemical signatures, which is usually achieved using chemometric methods.

Chemometrics is a highly powerful tool that uses mathematical and statistical techniques, such as multivariate analysis, to provide a researcher with the maximum relevant chemical information from complex data sets such as SERS spectra.^43^ Multivariate analytical techniques such as principal component analysis (PCA), partial least squares (PLS), linear discriminant analysis (LDA) and support vector machine (SVM) are discussed within this review. PCA is a descriptive technique that reduces the dimensionality of large datasets, increasing their interpretability through the creation of new, uncorrelated variables called principal components (PCs), whilst simultaneously minimising information loss and making it easier to identify variability.^44,45^ PLS regression differs from PCA in that it is a supervised learning method.^46^ It is a multivariate calibration technique that relates a set of independent variables *X* (for example, SERS intensity) to a set of dependent variables *Y* (for example, the concentration of a relevant biomarker). PLS projects the independent and dependent variables into sets of orthogonal variable scores so that the covariance between the two variables is maximised.^47^ On the other hand, LDA is a classification technique that uses information in a learning set of variables to construct a classifier that will separate predefined classes as much as possible.^48^ SVM, another supervised learning and classification technique used in the analysis of high dimensional data sets, compares data points on the basis of a kernel function, which is a metric of likeness between two points. The kernel function describes some feature space constructed from the data and the SVM optimises a separating hyperplane in the feature space.^49^

Strategies for the direct detection of microorganisms are highly desirable in the field because the sampling methodologies tend to be inexpensive and the detection range is not subject to the quantity, reliability, and specificity of biorecognition molecules traditionally used in label-based techniques such as antibodies and aptamers, meaning that they have the potential to have a shorter throughput time and be less operationally complex.

**Fig. 1:**
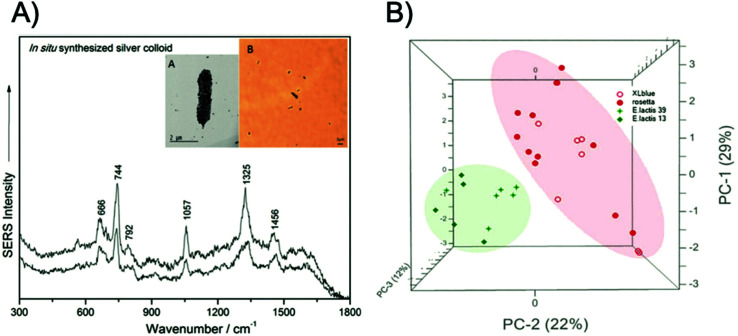
Single cell detection and discrimination of bacterial pathogens using a label-free SERS method. (A) Raw single-cell SERS spectra of the pathogen *E*. *coli* XL1-blue irradiated with a 633 nm laser wavelength, inset – transmission electron miscroscopy (TEM) micrograph (A) and 100× microscopy image (B) of *E*. *coli*, showing the *in situ* synthesised silver colloid coverage of the cell membrane. (B) PCA scores 3D plot showing the grouping of two Gram-positive (*E*. *lactis* CE13 and CE39, respectively) and two Gram-negative species (*E*. *coli* ROSETTA and *E*. *coli* XL1-blue). The images are reprinted from Dina *et al*.,^52^ Copyright (2017), with permission from Royal Society of Chemistry.

There have been numerous label-free approaches reported that demonstrate the multiplexed detection of pathogenic microorganisms. Zhang *et al.* demonstrated the use of magnetic-plasmonic Fe_3_O_4_@Au NPs for the concentration and detection of three Gram-negative bacterial strains, namely *Escherichia coli* (*E*. *coli*), *Acinetobacter calcoaceticus* (*A*. *calcoaceticus*) and *Pseudomonas aeruginosa* (*P*. *aeruginosa*), using a benchtop Raman system.^50^ In this study, a mixture containing bacteria with a concentration of 10^5^ colony forming units per mL (CFU mL^−1^) and NPs was dropped onto a silicon chip and condensed through the application of a point magnetic field. The three bacterial strains were identified and differentiated using PCA. In another approach, based upon the magnetic capture of bacterial cells, Wang *et al.* developed a three-step detection method called capture-enrichment-enhancement (CEE) using polyethyleneimine-modified gold coated magnetic microspheres and bimetallic gold/silver NPs.^51^ This method allowed for the collection and identification of bacteria in tap water and milk samples with an assay time of 10 minutes from incubation of the nanotags to SERS analysis using a portable Raman spectrometer. Furthermore, using PCA, *E*. *coli* and *Staphylococcus aureus* (*S*. *aureus*) were detected simultaneously at concentrations of 10^3^ CFU mL^−1^. Label free approaches have also been used to identify pathogenic microorganisms at the single cell level. Dina *et al.* designed a rapid and cost-effective technique for single cell label-free detection and species discrimination consisting of the *in situ* synthesis of silver NPs followed by analysis on Polysine™ adhesion microscope slides.^52^ Samples were analysed using a benchtop spectrometer coupled with an optical microscope that allowed for the spectra of individual NP encapsulated bacterial cells to be recorded. Single cell discrimination was then achieved between Gram-negative and Gram-positive bacteria, *E*. *coli*, *Morganella morganii* (*M*. *morganii*), *Enterococcus lactis* (*E*. *lactis*), and *Lactobacillus casei* (*L*. *casei*), in less than 5 minutes with a benchtop system, as is shown in Fig. 1.

Whilst label-free approaches do not depend on the efficacy of biorecognition molecules and can produce sensitive and specific pathogen detection, there are several challenges associated with their use. The first, and arguably the most important, is that it is difficult to obtain strong and reproducible SERS signals from pathogens and to consistently understand their biomolecular origins. It is crucial to ensure that SERS signals originate exclusively from the pathogens of interest within complex mixtures, because this will allow researchers to exploit label-free SERS multiplexing strategies more comprehensively and develop reliable, consistent diagnostic tests. Another challenge, pertaining primarily to bacterial detection, is that the SERS spectra of cells are likely to change due to external factors. SERS spectra provide information about cell wall structure, and hence spectral differences can be attributed to different biochemical components on the cell walls of different bacteria.^51^ External factors such as growth phase, nutrition supply and sterilisation protocols for growth media are likely to induce changes in the biochemical composition of the cell and hence cause differences in SERS spectra of different cells. Most label free approaches require benchtop spectrometers for analysis in order to achieve high detection sensitivity, which is compromised when a portable instrument is used.^51,53^ Furthermore, additional handling steps such as magnetic separation and filtration are required when portable instrumentation is used, leading to increased analysis time.^51,53^ Additionally, due to the complex nature of the SERS spectra obtained from pathogenic strains, it is difficult to visually discriminate between pathogens and hence all label-free biosensing requires data processing involving multivariate analysis in order to separate or confirm strain types. Chemometric approaches add a layer of complexity to detection platforms, although they are becoming more widely accessible with dramatic increases in computing power and machine learning knowledge.^54^ Alternative approaches involving the use of label-based SERS and microfluidics have been proposed to overcome some of these challenges and are discussed herein.

### 2.2. Microfluidics

Microfluidics is the control of the movement of fluids and the integration of sample preparation, reaction, separation and detection within a small portable device and is defined by the length scales at which laminar flow dominates, meaning that the physics of fluidic movement within such a device is changed from traditional fluid systems.^55^ Microfluidic devices can integrate various functions for high throughput and rapid analysis of very low volume fluid samples in the range of microlitres, especially when coupled with spectroscopic techniques such as SERS. Devices that have coupled microfluidics with SERS analysis have gained a lot of attention in the field of pathogenic microorganism identification because of advantages such as increased sample automation, integrated sample preparation, mixing and confinement, and increased portability. The combined strategies have high potential for translation into POU diagnostic applications.^56^

Microfluidic devices have been coupled with numerous label-based and label-free SERS techniques for the detection of pathogenic microorganisms. Wang *et al.* designed a low-cost multifunctional SERS chip that directly detected, as well as inactivated pathogenic bacteria at concentrations down to 100 CFU mL^−1^.^57^ The SERS chip was made of a silicon wafer decorated with silver NPs and modified with 4-mercaptophenylboronic acid (4-MPBA), a capture molecule that binds specifically and reversibly to peptidoglycan on the outer surface of bacteria.^58^ Furthermore, it was observed that the chip allowed for the spectral discrimination of *E*. *coli* and *S*. *aureus* in human blood samples with no spectral interference at 10^5^ CFU mL^−1^. Another label-free microfluidic platform was established by Hunter *et al.* who designed a reusable optofluidic platform containing a hollow-core photonic crystal fibre (HC-PFC) that provided a large SERS enhancement of bacteria cells when used in combination with silver NPs.^59^ This platform allowed for the simultaneous qualification and quantification of pathogens in serum and gave a comparable sensitivity to standard techniques such as PCR.^60^ The platform quantified and discriminated the pathogens *E*. *coli*, *S*. *aureus* and *P*. *aeruginosa* PAO1 using a genetically SVM learning algorithm at concentrations as low as 4 CFU mL^−1^ with a high accuracy and a 15 minutes analysis time.

With an aim to detect single bacterial cells using label-based SERS nanoprobes, Lin *et al.* integrated novel SERS active nanotags consisting of antibody and Raman reporter functionalised nanoaggregate-embedded beads (NAEBs) with a microfluidic nano-dielectrophoresis (nano-DEP) device for the capture of bacterial cells from solution.^61^ DEP is a technique that manipulates the motion of particles with electrical potential, such as cells, in a non-uniform electric field by creating a polarisability gradient between the particles and the surrounding medium.^62^ This strategy allowed for the capture and detection of *Salmonella enterica* (*S*. *enterica*) and *Neisseria lactamica* (*N*. *lactamica*) from dilute suspensions by analysing the SERS signal of NAEB-bacteria complexes and a limit of detection (LOD) of 70 CFU mL^−1^ was observed with a 10-minute measurement time. Discrimination of pathogenic and non-pathogenic strains was also realised with specificity at the single cell level. This assay showed the possibility of attaining a higher detection sensitivity than traditional techniques such as ELISA using a lower number of detection antibodies. Pazos-Perez *et al.* prepared a microorganism detection platform for the identification and quantification of the bacterial pathogens *S*. *aureus*, *E*. *coli* and *Streptococcus agalactiae* (*S*. *agalactiae*), on the millilitre scale and clinically relevant volumes of biofluids using antibody functionalised SERS-labelled silver NPs.^63,64^ Specific accumulation of the SERS tags on the pathogens within the fluid channel enabled multiplexed detection in serum without false positive identification and down to concentrations detectable using culture colony methods. The platform was then demonstrated using blood and it was found that the three pathogens could be detected with LODs in the tens of CFU mL^−1^, which is comparable to culture detection, in only 13 minutes as opposed to the 24 hour period required for culturing.

**Fig. 2:**
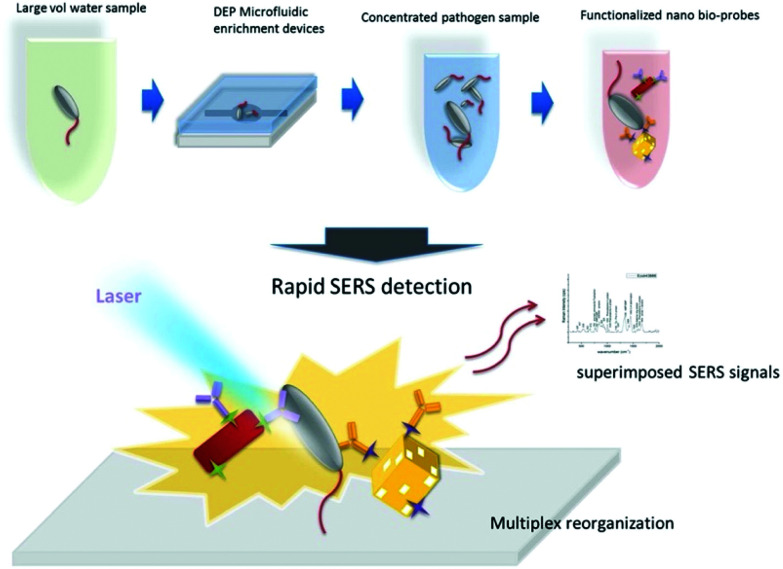
Schematic describing the rapid enrichment step using a microfluidic device and detection step using a multiplex self-referencing SERS strategy. Three anisotropic SERS tags, designed to target different epitopes on *E*. *coli* O157:H7 cells, were employed to yield a dual SERS signal containing contributions from the target pathogen and nanotags that confirmed specific pathogen recognition. The image is reprinted from Wang *et al.*,^65^ Copyright (2017), with permission from BioMed Central.

Wang *et al.* developed a ‘self-referencing’ portable biosensor, shown in Fig. 2, utilising multiple SERS probes and another nano-DEP microfluidic device for the detection and characterisation of *E*. *coli* O157:H7 at extremely low concentrations in water.^65^ The authors developed the concept of a self-referencing biosensor to mean one that achieves detection of a target pathogen in one single step brought about by a novel multiplexed targeting platform that utilises SERS molecular probes. The system was designed so that only specific binding of three unique anisotropic nanoprobes to different epitopes on a single cell would yield a detectable dual SERS signal comprising of a combination of three unique Raman reporters and the bacterial cell. Non-specific, or no binding, did not yield the dual signals because only the specialised SERS probes, which were designed with anisotropic NPs, could generate a sufficient SERS signal. The probes consisted of two gold nanorod (NR) tags functionalised with the Raman reporters 4-aminothiophenol (4-ATP) and 3-amino-1,2,3-triazole-5-thiol (ATT) and monoclonal antibodies specific to two different epitopes on *E*. *coli* O157:H7 cells. A third tag was prepared using a gold cage functionalised with an antibody specific to a third epitope and the Raman reporter 3-mercaptopropanoic acid (3-MPA). The three tags were deployed simultaneously to bacteria concentrated by DEP enrichment, and detection was achieved at the single cell level with sub-species specificity, which is highly desirable for frontline detection since an infectious dose of *E*. *coli* O157:H7 is as little as 0.2 CFU mL^−1^ in environmental samples.^66^ PCA was used along with a SVM learning algorithm to differentiate between pathogenic and non-pathogenic strains of *E*. *coli* with an accuracy of over 95%, even when the concentration of the pathogenic target was an order of magnitude less than the non-pathogenic control.

### 2.3. Nucleic acids

The detection of nucleic acid sequences that correlate to disease states and infectious pathogens is an incredibly important branch of diagnostics that offers a range of advantages including high sensitivity, the ability to quantify biological targets, minimal interference by antimicrobial agents, and a low limit of detection.^67^ The most commonly used DNA detection technique is PCR, which is often coupled with fluorescence based techniques such as nucleotide binding fluorogenic probes to monitor reaction times and as a confirmation of disease detection. Unlike fluorescence, SERS can be used to detect and analyse sequences pertaining to multiple disease targets simultaneously when it is coupled with DNA or RNA detection techniques. Additionally, nucleic acid-based biosensors can be developed for the indirect SERS detection of pathogenic microorganisms by coupling plasmonic substrates with Raman reporters and DNA or RNA ligands known as aptamers.^69^ Aptamers are developed from an *in vitro* selection process named Systemic Evolution of Ligands by Exponential Enrichment (SELEX) that optimises and selects nucleic acid sequences from a random sequence library in terms of their binding affinity to a given target.^70^ Aptamers have significant advantages over other targeting biomolecules such as antibodies, that include thermal stability, low susceptibility to denaturation, ease of production and low cost.^71^ There have been numerous SERS based sensing platforms reported for the identification and utilisation of nucleic acids with the goal of pathogenic microorganism detection.

Gracie *et al.* developed an assay for the detection and quantification of three bacterial pathogens prevalent in meningitis, *Haemophilus influenzae* (*H*. *influenzae*), *Streptococcus pneumoniae* (*S*. *pneumoniae*) *and Neisseria meningitidis* (*N*. *meningitidis*), using a combination of lambda exonuclease (λ-exonuclease) and SERS.^72^ DNA based assays are highly desirable for the identification of bacterial strains in suspected meningitis cases because immediate antibiotic therapy is required and this has the potential to alter the results of other testing platforms such as culturing. The assay involved the hybridisation of two complementary DNA probes, one of which contained a SERS active dye, to a target sequence followed by digestion of the double stranded hybridised DNA using λ-exonuclease and subsequent SERS detection of the digestion product. Synthetic target DNA sequences relating to the three meningitis pathogens were detected individually and quantified at concentrations in the picomolar (pM) range. Additionally, individual pathogen DNA quantification was achieved in a mixture using PCA and PLS regression with excellent predictability and reproducibility of the SERS signal intensity over multiple sample replicates and scans. This assay was later used for the positive detection of two meningitis strains using clinical samples from patients known to have meningitis indicating the potential of the platform to be used in a clinical environment.^73^

**Fig. 3:**
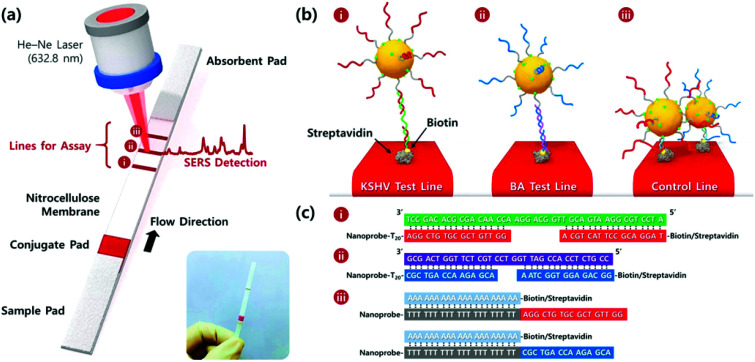
(a) Schematic illustration of the LFA biosensor for the simultaneous detection of two nucleic acids. The strip is composed of two test lines and one control line. (b) (i) KSHV DNA-AuNP conjugates were captured by the probe KSHV DNA on the first test line; (ii) BA DNA-AuNP conjugates were captured by the probe BA DNA on the second test line, and (iii) excess KSHV and BA detection DNA attached to the AuNPs were captured by control DNAs through T_20_-A_20_ hybridisation on the third control line. (c) Corresponding DNA hybridisations for two test lines (i and ii) and one control line (iii). The image is reprinted from Wang *et al*.,^74^ Copyright (2016), with permission from American Chemical Society.

Wang *et al.* developed a lateral flow assay (LFA) biosensor for the simultaneous detection of nucleic acids associated with Kaposi's sarcoma-associated herpesvirus (KSHV) and bacillary angiomatosis (BA) using SERS tags as shown in Fig. 3.^74^ KSHV is the leading cause of Kaposi's sarcoma (KS), which is one of the most prevalent cancers in the under-developed countries of Africa and is predominant in patients who are infected with human immunodeficiency virus (HIV).^75^ Rapid and effective clinical diagnosis of the two diseases is essential for patients in these countries because they have similar histopathological features and clinical presentations.^76^ The SERS tags were prepared by functionalising gold NPs with a Raman reporter and DNA probes complementary to part of the target viral DNA strands, which in this example were synthesised as oligonucleotide fragments. The LFA strips consisted of two streptavidin-biotinylated capture probes for KSHV and BA targets and a control line containing a DNA chain complementary to the spacer region on the DNA probes of both SERS tags. Peak intensities of the SERS tags on the test lines were used for quantitative analysis of both target DNA strains and detection was possible down to concentrations of 0.043 pM and 0.074 pM for KSHV and BA, respectively. The LOD concentrations observed indicated that the SERS LFA biosensor platform was 10 000 times more sensitive than a NP aggregation colorimetric LFA biosensor platform for the detection of KSHV, indicating the huge potential for SERS-based LFA platforms in the sensitive detection of DNA strains associated with viruses.^77^

Furthermore, Zhang *et al.* developed a SERS based sandwich assay for the simultaneous detection of the pathogens *Salmonella typhimurium* (*S*. *typhimurium*) and *S*. *aureus*, commonly associated with food borne diseases, using reporter probes prepared from NPs functionalised with different Raman reporters, and specific aptamers designed to target the pathogens.^78^ The sandwich assay was completed with the design of capture probes consisting of gold coated magnetic NPs functionalised with the same aptamers as the reporter probes. The two probes were used simultaneously to capture target bacteria in a reaction system and form a sandwich like detection structure that allowed for identification and quantification of the pathogens. Quantification of the two pathogens was achieved simultaneously from a mixture in the range between 10^2^ and 10^7^ CFU mL^−1^ and they were able to detect down to concentrations of 35 CFU mL^−1^ and 15 CFU mL^−1^ for *S*. *aureus* and *S*. *typhimurium*, respectively. In a study using another sandwich assay format, Zhang *et al.* observed the simultaneous detection and quantification of *E*. *coli* and *S*. *aureus* through dual recognition by broad spectrum vancomycin functionalised gold coated magnetic NPs and highly specific DNA aptamer functionalised SERS tags.^79^ The vancomycin functionalised magnetic NPs allowed for the capture of bacteria from complex sample mixtures within 15 minutes with efficiencies of 88.89% and 74.96% for *S*. *aureus* and *E*. *coli*, respectively. The selectivity of the system for the two target pathogens was tested and confirmed by introducing six other strains into the sample mixture and observing no interference in the SERS spectra. The target pathogens were also detected down to 20 and 50 CFU mL^−1^ for *S*. *aureus* and *E*. *coli*, respectfully, with logarithmic correlation between the bacterial concentration and the SERS intensity of the aptamer functionalised tags. The specificity and sensitivity of this platform was also proven in clinical samples. *E*. *coli* was detected and quantified in urine samples down to a concentration of 50 CFU mL^−1^ in 1.5 hours which is much more rapid than quantitative urine culture methods.^80^

Díaz-Amaya *et al.* prepared aptamer and Raman reporter functionalised gold NP SERS tags for the highly specific and sensitive detection of *E*. *coli* O157:H7 using a SERS assay that utilised the phenomenon of cell sedimentation of SERS tag-bacteria complexes within a food matrix and subsequent analysis of the residual supernatant.^81^ This assay format combined with the specific aptameric SERS tag allowed for the detection and quantification of target bacteria within 20 minutes in pure culture and highly complex ground beef samples down to concentrations of 10 CFU mL^−1^ and 100 CFU mL^−1^. The specificity of this platform was tested by comparing the target pathogen against *S*. *aureus*, *E*. *coli* B1201 and *E*. *coli* O55:H7 and observing that SERS signals obtained in the presence of interferent pathogens did not differ from controls performed without bacteria. Chen *et al.* fabricated an aptamer functionalised nano-popcorn plasmonic substrate for the sensitive and specific detection of influenzae A H1N1 in 20 minutes.^82^ Quantitative evaluation of the virus was confirmed by using the decrease of SERS intensity resulting from the release of the Raman reporter Cy3-labeled aptamer from the plasmonic surface *via* interaction of the virus and the aptamer. The specificity of the assay for influenzae A H1N1 was investigated and successfully confirmed by comparing the SERS response of the substrate against influenzae A H3N2, a 1 : 1 mixture of the two viral pathogens, bovine serum albumin (BSA), mucin and serum. The A H1N1 was also detected down to concentrations of 97 plaque forming units per mL (pfu mL^−1^), which was estimated to be 10 000 times more sensitive than the minimum concentrations detected using colorimetric ELISA assay results.

### 2.4. Immunoassays

An immunoassay is defined as a test for the quantitative determination of a biochemical target that utilises the specific binding between antigens and antibodies.^83^ Modern immunoassays incorporate the use of highly sophisticated platforms and are coupled with a variety of electrical and optical readout modalities such as colorimetry, fluorescence, surface plasmon resonance (SPR), fluorescence and SERS. Immunoassays are highly accurate and have been investigated robustly in disease diagnostics, environmental protection and food safety, and when coupled with SERS are able to produce readouts with extremely high sensitivity, specificity and a multiplexing capability that is not possible when using other readout platforms.^84,85^ It is for this reason that SERS based immunoassays have been developed and much success has been realised in the detection of pathogenic organisms using immunoassay platforms that are trending towards POU applications such as LFA and magnetic capture sandwich assays.

Sánchez-Purrà *et al.* created a multiplexing platform for the detection and discrimination of Zika and dengue non-structural protein (NS1) biomarkers using SERS active gold nanostars conjugated with specific antibodies for both targets and a dipstick immunoassay.^86^ It is difficult to distinguish between infections of Zika and dengue virus because of the overlap in clinical presentations, which consist of initial non-specific symptoms such as febrile illness, and geographic colocalisation in endemic areas because they share the same transmission vector of the *Aedes* genus mosquito.^87,88^ The dipstick consisted of a LFA strip onto which Zika and dengue NS1 antibodies, equivalent to the antibodies conjugated to the SERS tags, were immobilised on the test line. Nanotags were mixed with Zika and dengue NS1 in human serum and the dipstick was submerged in this mixture. The ability of each antibody pair to detect the NS1 antigens was investigated using colorimetric tests and SERS readouts. The antigens were detected down to concentrations of 0.72 nanograms per mL (ng mL^−1^) for Zika NS1 and 7.67 ng mL^−1^ for dengue NS1 using SERS, corresponding to 15 and 7-fold lower detection limits than those observed using colorimetric readouts. Wang *et al.* developed a quantitative SERS based LFA strip for the simultaneous detection of influenza A H1N1 virus and human adenovirus (HAdV) with a 30 minutes analysis time by using antibody conjugated SERS nanotags with silver coated magnetic NP cores.^89^ The nanotags were added to viral samples and after incubation were captured by magnetic separation. Nanotag-virus complexes were then resuspended and introduced to the LFA strip, which consisted of two test lines immobilised with H1N1 and HAdV specific detection antibodies and a control line. Therefore, magnetic SERS tag-virus-detection antibody sandwich complexes were formed on the test lines when specific binding took place, confirming specific capture of the viral pathogens from solution. The specificity of the platform was confirmed through colorimetric identification and SERS analysis on the test lines, and the pathogens were quantified by analysing the SERS peak intensities obtained from the nanotags in the sandwich complex. Concentration studies confirmed that the viruses could be detected down to 50 and 10 pfu mL^−1^ for H1N1 and HAdV, respectively, indicating that the assay was capable of a LOD 2000-fold lower than a commercially available colloidal gold colorimetric assay and 100-fold lower than ELISA for the detection of the same viruses. Furthermore, the accuracy and practicality of the assay was evaluated by conducting controlled experiments in biological samples, such as whole blood, serum, and sputum, spiked with 10^5^ pfu mL^−1^ samples of H1N1 and HAdV. The results of these tests confirmed that the magnetic SERS LFA strip could be utilised for virus detection in complex samples with high specificity.

**Fig. 4:**
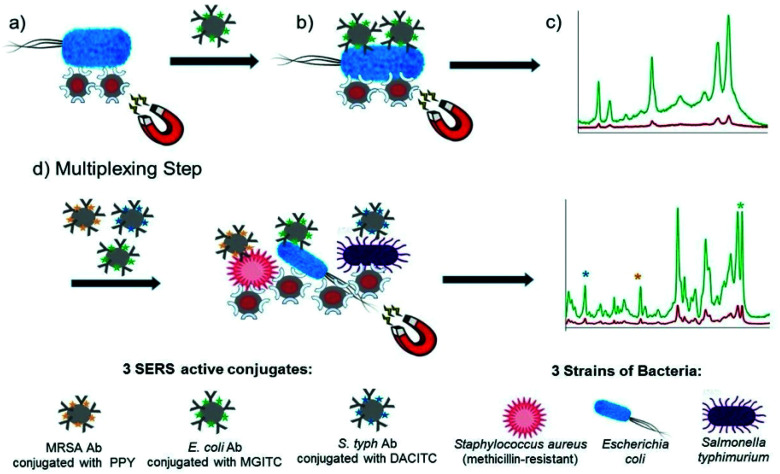
Schematic illustrating the single-plex and multiplex detection assay. Assay format: (a) lectin (Con A) functionalised silver coated magnetic nanoparticles (Ag@MNPs) will bind to bacteria and the presence of the magnet will allow for magnetic separation of the bacteria from the sample matrix (b) SERS active silver nanoparticles (AgNPs) functionalised with a biorecognition molecule (antibody; Ab) and a unique SERS reporter are added. The mixture is shaken for 30 min before applying a magnet for a further 30 min and allowing the sample to collect. Any unbound matrix is gently removed, and the sample subsequently re-suspended in dH_2_O (c) The sample is then interrogated with a 532 nm laser beam and SERS signal obtained (green spectrum). When no target is present the functionalised AgNPs will be washed away, thus they will not bind to bacteria, so a minimum SERS signal obtained (red spectrum). (d) Multiplexing step: 3× AgNP conjugates each functionalised with a different Raman reporter and an antibody (which is specific for a bacterial pathogen) are added together with 3 bacterial pathogens and Con A (which binds to all three bacteria) functionalised Ag@MNPs. In the same way as the single-plex systems magnetic separation allows for the samples to be concentrated and analysed *via* a 532 nm laser. A SERS spectrum is obtained which contains characteristic peaks from the three Raman reporters and thus can be used to confirm if the targets are present. The image is reprinted from Kearns *et al.*,^90^ Copyright (2017), with permission from American Chemical Society.

Kearns *et al.* reported the simultaneous detection of three bacterial pathogens, *E*. *coli*, *S*. *aureus* and *S*. *typhimurium*, with high selectivity and sensitivity using a magnetic sandwich assay incorporating antibody functionalised SERS reporter NPs and lectin functionalised magnetic NPs.^90^ The assay, illustrated in Fig. 4, involved the broadband capture and isolation of bacteria from a sample matrix using the lectin functionalised magnetic NPs followed by their detection using the strain specific antibody functionalised SERS active NPs. The pathogens were subsequently captured using a magnetic plug and interrogated with a laser allowing their simple and rapid optical detection. The three target pathogens were all isolated and detected down to extremely low concentrations of 10 CFU mL^−1^ individually. In addition, a mixture containing three pathogens was detected and discriminated from three individual samples at 10^3^ CFU mL^−1^ using PCA. This assay format has since been transferred to a portable system, as will be discussed in the POU section, demonstrating its potential as a rapid, reliable on-site diagnostic tool for bacterial pathogens. Neng *et al.* simultaneously detected trace quantities of multiple viral and bacterial pathogens, West Nile virus (WNV), Rift Valley fever virus (RVFV) and *Yersinia pestis* (*Y*. *pestis*), in serum using a SERS based sandwich immunoassay involving silica-encapsulated SERS NPs specific to each antigen and magnetic NPs conjugated with antibodies.^91^ Each SERS tag was mixed with 20% fetal bovine serum (FBS) containing three pathogen antigens and capture magnetic NPs and the resulting immunocomplexes were separated from the serum using magnetic capture and analysed using a handheld Raman device. Silica was used as a protective shell to encapsulate the SERS active NPs because it renders them impervious to matrix and ionic effects, which is extremely useful for detection of pathogens in complex solutions. All reporters and hence antigens were detected visually at concentrations of 10 pg mL^−1^ with no sample preparation of the serum solution required.

This section has clearly demonstrated the huge effort that has been made over the past decade to advance the research into pathogen detection *via* SERS. However, for these SERS platforms to be used at the forefront of science, they need to be reliable, rapid, specific, sensitive, user-friendly, and field deployable. The next section will discuss the recent literature on POU applications and the prospects of these systems for use in biomedical and industrial settings.

## 3. Point of use SERS multiplexing approaches

POU approaches to pathogen detection are essential for translating techniques such as SERS into settings where on-site analysis is required such as medical centres, food production sites, environmental monitoring and bioterrorism.^92,93^ However, in order to be seriously considered in these settings, POU SERS assays and devices need to be robust, rapid, sensitive, specific, cost effective, reproducible, user friendly, adaptable and field deployable, as stipulated by the World Health Organisation (WHO).^94,95^ POU tests for the identification of pathogenic microorganisms are designed to be performed by non-laboratory trained individuals such as medical, industrial and security staff to inform them of necessary preventative measures, and the main challenges associated with these tests are the intrinsic capability of the technologies and quality assurance for the end-users to ensure the accuracy of results.^96^ Significant progress has been made in translating the technologies discussed in the previous section into potential SERS POU applications through testing on real clinical or environmental samples and transitioning from benchtop to more portable spectrometers. This section will highlight examples from the four SERS approaches discussed in the previous section that have taken important steps towards platforms that can support label-free and label-based multiplexed pathogen detection in POU contexts and settings, and the future steps needed for each approach to better satisfy the requirements of POU testing.

### 3.1. Label free approaches

Label free SERS techniques have been realised for the POU multiplexed detection of pathogens in both medical and industrial settings. Wu *et al.* developed a label free, deployable SERS assay for the detection and differentiation of foodborne pathogenic bacteria and confirmed the deployability of the assay against infected mung bean sprouts.^53^ The assay utilised vancomycin functionalised silver nanorod array substrates in combination with both portable and handheld Raman systems for the quantitative and sensitive detection of six common foodborne pathogens. The food samples were filtered in a two-step process involving a crude pre-filtration followed by a second filtration to remove target bacteria and incubation with SERS substrates for analysis. The whole assay time from the filtration of the infected mung bean sprouts to SERS analysis was 4 hours, which is significantly faster than traditional methods. The six strains were detected down to concentrations of 10^2^ CFU mL^−1^ in solution or 10^3^ CFU g^−1^ of mung bean sprouts and discriminated using the chemometric techniques PCA and PLS regression at the species and strain level. This study demonstrated quantitative, field deployable detection of multiple pathogens simultaneously using portable and handheld systems, but its translation into an industrial setting may still be obstructed by the need for an extra filtration step that is not integrated into the detection platform and chemometrics. Furthermore, for this technology to be used at scale in a food production setting, the analysis time and sensitivity of the detection platform would likely have to be improved when detecting real food samples. Infectious doses of the bacteria *S*. *enteritidis* and *E*. *coli* O157 in food samples have been previously reported as less than 30 and 9 CFU g^−1^, respectively, and 4 hours is not sufficiently rapid for large scale testing in an industrial context.^97^ Mungroo *et al.* detected eight foodborne pathogens using a SERS based microfluidic chip in combination with chemometric analysis.^98^ The microfluidic device consisted of two inlets for bacterial suspensions and silver NPs, a sensing window for SERS measurements and an outlet connected to a capillary pump. Bacteria and NP suspensions were pipetted into the device and measurements were made on a portable system. Spectra were then analysed using PCA and LDA to allow for discrimination and classification of eight foodborne bacteria. This approach allowed for discrimination assignment of bacterial SERS bands for each pathogen, in various combinations of polymicrobial mixtures, with a low-cost device that has a high potential for on-site use, and detection was observed at highly relevant concentrations for all pathogens down to a LOD of 2 CFU mL^−1^ for a polymicrobial mixture of *E*. *coli* and *P*. *aeruginosa*. However, the chemometric techniques involved mean that there are additional layers of complexity involved in the technique when compared to visual discrimination of the Raman signatures and the prospects of this approach would hinge on the integration of data processing into the system to increase its usability by non-experts in the field. Like the previous example, the next steps for this detection platform should involve testing and optimisation of the technique with real infected food samples with necessary sample preparation in resource limited settings, with a focus on sensitivity for all target pathogens, specificity which is hugely challenging when aiming to detect such diverse sample mixtures, and rapidity of detection.

**Fig. 5:**
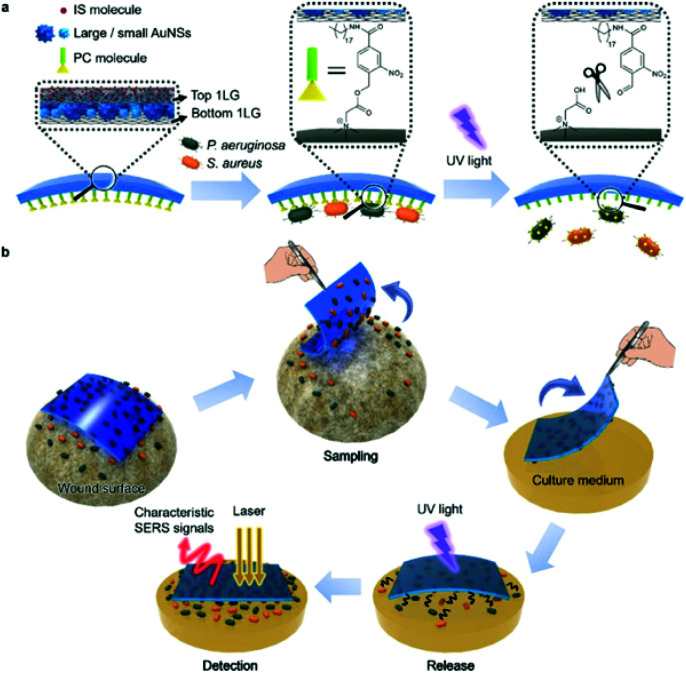
Schematic illustration of (a) SERS adhesive tape for pathogen capture and release and (b) pathogen sampling from skin wound, photo-controlled release to solid culture medium for pathogen growth, and *in situ* SERS detection of *S*. *aureus* and *P*. *aeruginosa*. The image is reprinted from Guo *et al.*,^99^ Copyright (2019), with permission from American Chemical Society.

Guo *et al.* designed an intelligent adhesive tape, depicted in Fig. 5, as a “three in one” platform for rapid sampling (5 minutes), photo-controlled release and SERS detection of *S*. *aureus* and *P*. *aeruginosa* from infected wounds.^99^ The user-friendly tape was prepared by encapsulating densely packed multi-sized gold nanostars as SERS substrates between two pieces of graphene and modifying the surface with an *o*-nitrobenzyl derivative to form an interface for pathogen capture with a UV-responsive cleavable section that allowed pathogens to remain viable after release onto solid culture medium for growth and SERS detection.^100,101^ To mimic the early stage of concurrent wound infections, a 10^6^ CFU mL^−1^ mixture of *P*. *aeruginosa* and *S*. *aureus* was spiked onto a skin burn wound of a mouse, then sampling, transfer and release for growth, and SERS detection were performed with the adhesive tape. After the controlled release of pathogens and 8 hours of growth on Luria–Bertani (LB) agar, the specific SERS signals for each pathogen were observed and the respective distribution of each pathogen on the culture medium was obtained by SERS imaging. This is a highly promising, convenient, and efficient method of pathogen detection in skin wounds, with a user friendly and field deployable sampling method that has been demonstrated on an artificially infected sample. However, it requires the use of highly specialised equipment such as a confocal Raman microscope that is unsuitable for POU testing and only trained personnel can operate, and this would most likely prevent its transition into a POU detection strategy unless the sampling tape could be used in combination with a portable or handheld system. Additionally, whilst the sampling method was rapid, the pathogens were cultured for 8 hours prior to SERS detection. In a clinical setting infections can progress significantly within that time frame, meaning that the overall analysis time of the platform is not yet sufficiently rapid to accurately prescribe antibiotic doses for a patient's infection levels.^102^ The prospects of the technology also depends on the successful capture and detection of pathogens at lower, more clinically relevant concentrations to mimic early diagnosis of wound infections and further optimisation on real infected samples and a wider range of pathogenic strains.

Kamińska *et al.* detected and discriminated between three meningitis pathogens, *N*. *meningitidis*, *H*. *influenzae* and *S*. *pneumoniae*, in clinical cerebrospinal fluid (CSF) samples using a label free SERS detection assay and PCA.^103^ Lumbar puncture is commonly used in combination with clinical features for meningitis diagnosis because the CSF can provide clinicians with large amounts of information that enable conformation of infection through the identification of human biomarkers and the direct detection of pathogens using traditional methods.^104^ The detection assay is based on the combination of two types of colloidal gold and silver-coated nucleopore track-etched polycarbonate membranes that allow for simultaneous filtration of CSF, immobilisation of CSF components including leukocytes, albumins, globulins and pathogenic microorganism, and the enhancement of their Raman signals. Several spectra were recorded from 48 clinical samples, 38 of which were from patients who had tested positive for meningococcal meningitis, and positive identification of the three bacterial strains was confirmed against the negative control sample group and against spectra collected of the strains directly on solid culture media. These results represent the controlled isolation, analysis, and label-free discrimination of multiple bacterial strains from highly complex clinical fluids. Analysis of the bacteria functionalised membranes was completed on a confocal Raman microscope which is unsuitable in a clinical environment, but the setup for the injection of clinical fluids into the SERS active membranes was portable and could be easily transferrable to the field. Optimisation of the membrane filtration system with a smaller, more portable, Raman spectrometer would be beneficial for bringing the detection platform closer to a clinical setting, as well as the demonstration of quantitative analysis at clinically relevant concentrations of bacteria in CSF samples. In the same study, the detection of neopterin, a diagnostic marker used in the determination of bacterial meningitis infections, was demonstrated by comparing SERS spectra of control and positive CSF samples.^105^ Neopterin concentration was found to be significantly higher in infected CSF samples and the SERS study used produced results comparable to ELISA.

**Fig. 6:**
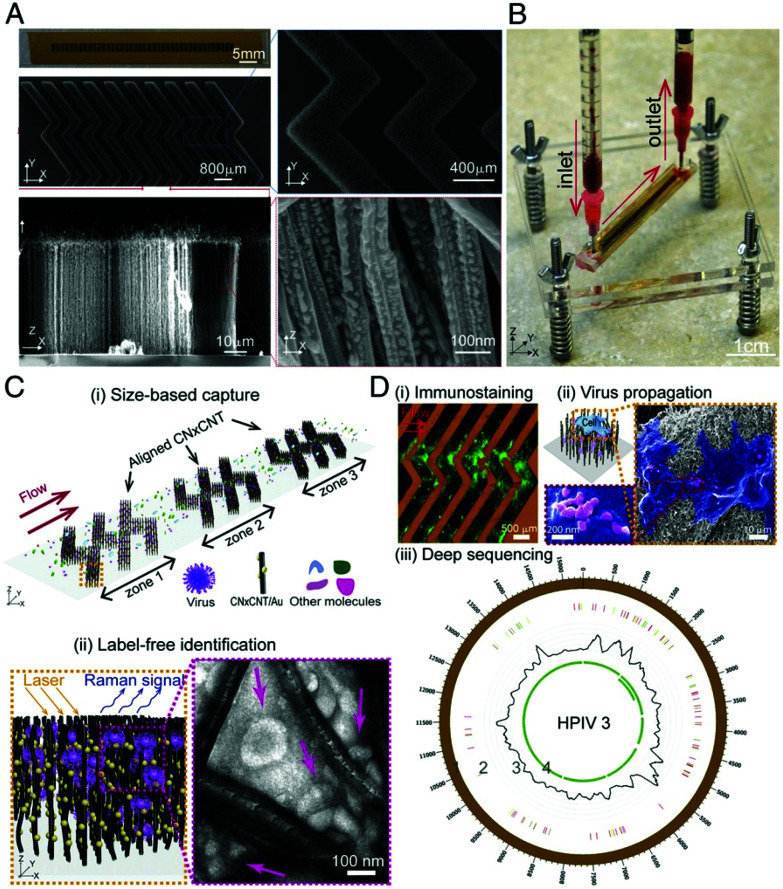
Design and working principle of VIRRION for effective virus capture and identification. (A) Photograph and SEM images of aligned CNTs exhibiting herringbone patterns decorated with gold nanoparticles. (B) Picture showing assembled VIRRION device, processing a blood sample. (C) Illustration of (i) size-based capture and (ii) *in situ* Raman spectroscopy for label-free optical virus identification. Images of electron microscopy showing captured avian influenza virus H5N2 by CNxCNT arrays. (D) On-chip virus analysis and enrichment for next generation sequencing, (i) on-chip immunostaining for captured H5N2, (ii) on-chip viral propagation through cell culture, and (iii) genomic sequencing and analysis of human parainfluenza virus type 3 (HPIV 3). The image is reprinted from Yeh *et al.*,^106^ Copyright (2019), with permission National Academy of Sciences.

Yeh *et al.* developed a rapid, portable and label free platform, called VIRRION (virus capture with rapid Raman spectroscopy detection and identification), for multi-virus capture and identification from clinical samples with minimal preparation.^106^ VIRRION consists of a handheld microfluidic device, illustrated in Fig. 6, designed to simultaneously capture viruses of different sizes using aligned nitrogen doped carbon nanotubes (CNxCNTs) decorated with gold NPs while preserving their structural integrity and viability, to perform real-time non-destructive identification using SERS coupled to a machine learning algorithm and spectral database. Gold NP decorated CNxCNTs were patterned within herringbone arrays with tuneable intertubular distances (ITD) for the capture of viruses sized between 22 and 720 nm.^107^ To test for *in situ* optical identification of multiple virus particles within the CNTs using SERS, spectra were collected for influenza A H5N2, influenza A H7N2 and reovirus. The spectral fingerprint for each virus was distinguishable at low concentrations, comparable to PCR, using PCA. VIRRION was also validated in human respiratory infection diagnostics by rapidly capturing different viruses in nasopharyngeal swabs from patients who had previously been diagnosed with rhinovirus, influenza A virus or human parainfluenza virus type 3 (HPIV). A VIRRION system was assembled with ITDs of 200, 100 and 30 nm which cover the size range of most viruses known to cause respiratory infections. The SERS fingerprints of each virus was used with PCA to demonstrate that the Raman spectra could be used to clearly differentiate between the strains. VIRRION has demonstrated the efficacy of techniques that couple highly reproducible SERS substrates with machine learning algorithms, and the future development of the platform will involve the expansion of the Raman database with the ultimate end goal of viral strain prediction.

### 3.2. Label based approaches

Label based SERS techniques have also gained traction in the POU multiplexed detection of viral and bacterial pathogens. In a continuation of previous work, Kearns *et al.* transferred a novel magnetic solution assay for the sensitive and specific capture of detection of bacteria to a portable SERS system and demonstrated its potential as a reliable on-site diagnostic platform.^108^ Initial tests validated the ability of the assay, involving lectin functionalised magnetic NPs for capture followed by specific detection using SERS active antibody functionalised NPs, to identify *E*. *coli* and *S*. *aureus* down to concentrations of 10 CFU mL^−1^ with high reproducibility in the SERS intensity, demonstrated by a low relative standard deviation over a large number of scans.^90^ The system was also used to discriminate between the two strains within a mixture at concentrations of 10^3^ CFU mL^−1^ using PCA with an analysis time of 1 hour, although visual discrimination was also possible. To make this platform more transferrable to on-site diagnostic applications, future work will involve simplification of sample preparation for non-laboratory-based end-users, attempting to decrease the analysis time to ensure that the platform could be scaled up, making the system more user friendly and testing of more complex clinical and food specimens. In another example of the progression of a previously reported technique into a POU application, Gracie *et al.* demonstrated the detection of *S*. *pneumoniae* and *N*. *meningitidis* genomic DNA extracted from the CSF and blood of hospital patients admitted for sepsis and meningitis using a SERS assay and confirmed its potential for use in hospital laboratories.^72,73^ It should be noted that it is significantly more difficult to detect whole genomic DNA sequences extracted from pathogenic cells than it is to detect oligonucleotides synthesised to mimic the same genomic sequence, especially in a clinical setting where the pathogens are suspended in biofluids.^109^ The study, which involved a significantly shorter analysis time than traditional culture-based techniques (5 hours *versus* 36 hours for culture based techniques), detected the two pathogens individually and simultaneously with equally successful results in clinical samples of patients with confirmed bacterial meningitis. From the 28 clinical samples provided for the study, 9 gave excellent spectral discrimination by eye to allow for pathogen identification using the SERS bands of the reporter probes in the assay. There are still significant challenges to be addressed in transitioning the technique into a POU testing platform, such as reducing the detection time, integrating sample preparation of the clinical fluids, and testing on much larger clinical sample pools where the diagnosis is not known beforehand. This assay is not limited to the detection of bacterial meningitis pathogens, and it has a high potential for the detection of a variety of infectious diseases in a clinical environment because the sequences of the capture and reporter probes can easily be changed depending upon the target pathogen.

As previously discussed, LF coupled with SERS detection is a technique that has an extremely high potential for POU diagnostics in a medical setting. In a study highlighting this high potential, Zhang *et al.* developed a 2 × 3 microarray on a LF strip with SERS nanotags encoding the nucleic acids of 11 common respiratory tract infection (RTI) pathogens and observed the detection of different concentrations of synthetic oligonucleotides inoculated into a blank throat swab.^110^ In this study, 11 capture nucleic acids corresponding to the pathogens, both bacterial and viral, were incorporated into the microarray consisting of six test dots on the nitrocellulose membrane with each one of the five dots detecting two nucleic acids and one dot detecting just one. For the test dots with two capture nucleic acids present, nanotags consisting of silver core gold shell NPs and two distinct Raman dyes were designed. The nanotags were placed on a conjugate pad for incubation with target nucleic acids from a sample solution and the nanotag-target conjugates were then detected both visually and using SERS analysis of the test dots. The results obtained confirmed that this platform could detect pathogenic nucleic acids rapidly with ultrahigh sensitivity and no cross reactivity. All the nucleic acid targets could be detected down to concentrations 2 orders of magnitude lower than 1 pM, and quantification was possible between 1 pM and 50 nM. Furthermore, the time taken for the whole test to be completed was 20 minutes, including 7 minutes for the reaction to take place and 13 minutes for Raman signals to be obtained for each test dot. Many aspects of this testing platform make it compatible with the requirements of POU testing, such as the portability of the LF strips, the rapidity of the overall test, and the sensitivity and specificity of the SERS nanotags used. However, despite the clear advantages of this technique, it should be noted that testing was completed on synthetic DNA fragments artificially incorporated into blank throat swabs, which are significantly different to genomic DNA extracted from pathogenic cells. To be considered as a testing platform for RTI pathogens in a resource limited setting, the detection platform would have to be applied to whole pathogen genomes inoculated into blank throat swabs and eventually on real clinical samples.

Liu *et al.* also demonstrated the POU potential of SERS based LF detection by combining the technique with recombinase polymerase amplification (RPA) for the simultaneous detection of *Listeria monocytogenes* (*L*. *monocytogenes*) and *S*. *enterica* in real food samples such as milk, chicken breast and beef.^111^ RPA is an isothermal nucleic acid amplification technique that uses enzymes to separate double stranded DNA, assist in primer and target recognition and primer extension.^112^ The low temperature requirements make it an excellent DNA detection technique in the field, and it is often used in combination with fluorescence and naked eye strategies such as gel electrophoresis.^113^ Whilst these techniques are useful, they are not suitable for multiplex RPA assays which are rarely reported. For this assay, DNA primers were designed based on the two individual strains and they were tagged with organic compounds for antibody capture and biotin. Furthermore, SERS tags consisting of Raman reporter capped gold/silver core–shell NPs conjugated with streptavidin were prepared for conjugation to the primers, and a mixture of primers and SERS tags were deposited on the sample pad of a LF strip containing two test lines with antibodies specific to the primer labels and a control line with anti-streptavidin antibody. The specificity and sensitivity of the assay was investigated by performing the assay with genomic DNA of multiple different strains and by conducting concentration studies for the two target strains. Visual detection was achieved within 30 minutes down to concentrations of 190 and 270 CFU mL^−1^ for *L*. *monocytogenes* and *S*. *enterica*, respectively, and the limit of detection using SERS analysis of the Raman reporter on the SERS tag was 2 orders of magnitude lower. Furthermore, when the bacteria were harvested from real samples of milk, chicken and beef, the limits of detection for both strains were between 22 and 35 CFU mL^−1^. Although the SERS analysis, which involved analysis of the test lines using a Raman microscope, adds more operational complexity to the assay when compared to naked eye based LF detection, quantification is possible, and the sensitivity of detection is significantly improved. The system could be improved in a POU context by conducting analysis of the test lines on portable or even handheld Raman spectrometers. This would still allow for the advantages associated with the SERS analysis over visual detection to be retained whilst also improving the portability of the assay in resource limited settings. The sample preparation required in this detection platform is also highly complex, and significant preparation is needed to obtain the bacterial DNA for the final LF-SERS detection assay because in its current form it is unlikely that this assay could be performed by an end user who is not proficient in the numerous laboratory techniques involved.

**Fig. 7:**
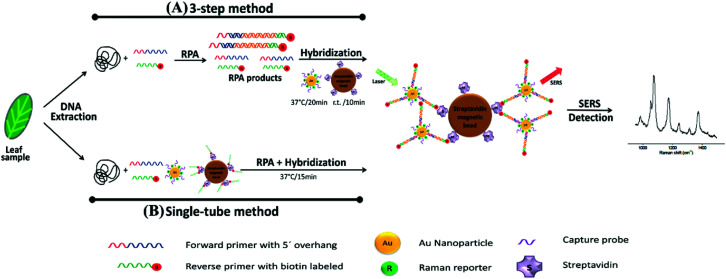
Schematic illustration of RPA/SERS assay. (A) 3-Step method consisting of separate DNA amplification, hybridisation, and detection. Briefly, genomic DNA was extracted from plant tissue, followed by RPA to amplify unique genomic regions of each pathogen using specific primer sets. The primers were designed such that RPA products would contain a biotin handle on one end and a 5′ overhang sequence on the opposite end, which functioned as a barcode for hybridising to SERS nanotags containing Raman reporters and complementary capture DNA. After amplification, the biotin/RPA/SERS products were captured by streptavidin magnetic beads for SERS detection. (B) Single tube method that condensed the amplification, hybridisation, and magnetic capture of RPA products into a single reaction step. The image is reprinted from Lau *et al.*,^114^ Copyright (2016), with permission from American Chemical Society.

The combination of SERS and RPA was also utilised for the multiplexed POU detection of plant pathogens. In this study, Lau *et al.* demonstrated the multiplexed detection of three agriculturally important plant pathogens, *Fusarium oxysporum* (*F*. *oxysporum*), *Botrytis cinerea* (*B*. *cinerea*) and *Pseudomonas syringae* (*P*. *syringae*), in complex systems such as commercial tomato crops in the field.^114^ Plant leaves were infected with five common agricultural pathogens and sampling was completed in such a way that different leaves displayed various degrees of symptom severity to create a scoring system. A schematic summarising the assay is shown in Fig. 7. Genomic DNA was extracted from the leaves and RPA was performed to amplify unique genomic regions of each pathogen using specific primer sets that were designed such that RPA products would contain a biotin handle on one end and a 5′ overhang sequence on the opposite end for functionalisation to strain specific SERS tags. After amplification, biotin-RPA-SERS conjugates were captured by streptavidin magnetic beads and Raman signals of the nanotags were analysed using a portable spectrometer. The SERS and RPA assay was 10^2^ and 10^4^ times more sensitive than RPA and PCR alone, and it was demonstrated that infection was detected for multiple pathogens simultaneously on commercial tomato leaves that scored low for symptom severity. Additionally, the assay was performed outside of the lab in a single tube RPA/SERS assay for the three pathogens simultaneously, halving analysis times needed (40 minutes using the single tube variation) to perform three separate assays. It is clear from the current and highly promising results that this RPA-SERS platform could be applied to a wide variety of agriculturally relevant pathogens, and testing that involved multiplexed detection and quantification of pathogens outside of the laboratory on real samples would be highly advantageous for the future development of this technology. Furthermore, like the previous example, the sample preparation required is currently a limiting factor in this technique's wider development, and any future iterations of this technology would benefit from an integrated, user friendly protocol that reduces sample handling.

**Fig. 8:**
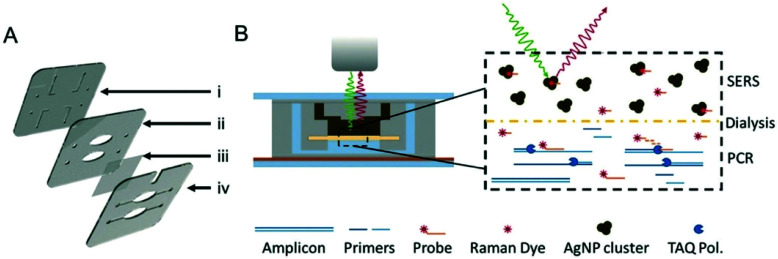
Illustrations of dialysis driven PCR-device. (A) Rendering of an exploded 3-layer PCR-SERS device. The top layer (i) contains inlets and outlets as well as channels for the SERS well in the second layer (ii). A dialysis membrane (iii) is embedded between the middle and bottom layers (iv) to separate the SERS well from the PCR wells in the bottom layer. (B) Schematic of device function during a probe-based qPCR assay, in which hybridisation of a dye labelled probe is degraded during extension of the PCR primer by Taq polymerase. Degradation of the probe liberates the dye, which can freely pass-through membrane pores (20 kDa cut-off) to the AgNP colloid for SERS detection. The image is reprinted from Restaino and White,^115^ Copyright (2018), with permission from Royal Society of Chemistry.

SERS has also been coupled with PCR to provide multiplexed optical readouts following DNA amplification. Restaino and White detailed a real-time, low-cost PCR-SERS thermoplastic microsystem that allowed for the simultaneous amplification of nucleic acids from two methicillin-resistant *Staphylococcus aureus* (MRSA) biomarking genes and product separation into a SERS active silver colloid for real time detection.^115^ An illustration of the dialysis driven PCR-device is shown in Fig. 8. Miniaturisation of existing laboratory systems such as PCR to provide mobile versions in the field is a promising path for POU diagnostics, and the combination with SERS allows for the simultaneous detection of multiple DNA targets, thus reducing time and costs. In this platform, a dialysis membrane was used to isolate the solution stable silver colloid aggregates from the PCR reaction that continuously released Raman reporters specific to the individual genes. The detection of the Raman reporters thus indicated positive amplification of target genes. In this study, MRSA genes *MecA* and *FemA*, which are used to identify *S*. *aureus* and methicillin resistance, were modified with Raman reporters and these samples were loaded into a PCR well separated from an upper well containing silver colloid by the dialysis membrane.^116^ The microchips were then sealed and placed in a custom thermocycler in which thermal cycles and spectrometer readings were synchronised. SERS data was collected at the end of every reaction cycle and signal intensity was measured at the height of one of the Raman reporter specific peaks to monitor PCR. Monitoring of SERS spectra indicated positive amplification of both target MRSA genes in both independent and combined primer sets which were all distinct from negative controls. Whilst this example was an early demonstration of multiplexed PCR with SERS, the device could be tested in the future on a much wider library of genes and dyes in a single well, and integrated sample preparation should also be a consideration when transitioning to the analysis of genomic DNA directly from pathogenic cells.

## 4. Conclusion and future perspective

Most of the papers discussed in this review are excellent examples of how the bioanalytical and spectroscopic fields are taking critical steps towards novel SERS systems that can support sensitive and reliable pathogenic detection in the field. Researchers have utilised a wide range of techniques to achieve this feat that span multiple scientific disciplines, from label-free detection platforms that apply chemometrics, machine learning and microfluidic devices to label-based methods that make use of antibody and aptamer functionalised SERS nanotags for nucleic acid detection, magnetic capture assays and LFA. However, each POU device discussed currently has limitations such as requiring trained personnel to carry out analysis, specialised equipment, complex data processing or the limits of detection are out with safety standards which is preventing them being used in industrial and medical settings. Some of these challenges are more relevant to label-free or label-based devices and assays because, as discussed throughout the review, they have different requirements and end goals for pathogenic microorganism detection. For example, many of the label-free and microfluidic approaches are currently limited by the need to perform chemometrics on acquired spectra, which require skilled personnel to perform or large data libraries for machine learning algorithms, although they have the potential for automation. On the contrary, many of the label-based techniques discussed allow for visual discrimination, but they require complex sample preparation, for both test reagents and samples, that is simply unsuitable for targeted end users to perform in resource limited settings. Every device and assay presented in this review has its own unique advantages and challenges, and any future success in transitioning from the laboratory bench to the market will depend largely on the ability of the researchers to ensure that their detection strategies meet all the requirements of POU testing and can be proven to be robust, rapid, sensitive, specific, cost-effective, reproducible, user-friendly, adaptable, and field deployable.

Delivering SERS to the market has thus far mainly focussed on the development of commercially available SERS active substrates and nanoparticles. One of the first commercially available SERS assays was developed by the authors, through Renishaw Diagnostics, for the multiplexed detection of fungal infections using a DNA based approach which was CE marked but not continued beyond 2016 for commercial reasons. Other SERS based assays have been investigated by major companies and are expected to be widely available in the near future. For example, Becton Dickinson have developed a no wash SERS based assay to distinguish Ebola from malaria or Lassa.^117^ There are multiple approaches currently under development for commercialisation, harnessing the benefit of SERS for bioanalytical applications, in particular the multiplexing capability of the approach and we fully expect to see more SERS based approaches enter the market in the future.

A major issue in preventing the transition of the discussed platforms into POU detection systems is the limited testing conducted on real-world samples. Hence, it is essential that multidisciplinary collaborations are established, with contributions from biologists, chemists, physicists, and engineers, to produce comprehensive testing devices that integrate sample preparation, spectral acquisition, and analysis, and that the innovative POU devices are tested robustly in ‘realistic’ settings (such as food production sites, water treatment facilities and medical centres) out with highly controlled laboratory environments. By doing so, together we can overcome current limitations with testing devices whilst working on real-life samples and have SERS devices and detection systems which are rapid, user-friendly, cost effective and reliable. The unprecedented events experienced worldwide throughout 2020 have reintroduced the threat of pathogenic microorganisms to the public. Novel influenza outbreaks and antimicrobial resistance are now statistical certainties and are not a matter of “if”, but “when” and “how serious”. It is essential for academia, industry, business, funding bodies and governments to be prepared for these inevitable outcomes, and they must come together and work in cooperation to save lives and prevent the uncontrolled outbreak of these potentially devastating infectious diseases.

## Author contributions

The manuscript was written through contributions of all authors and all authors have given approval to the final version of the manuscript.

## Conflicts of interest

The authors declare no competing financial interest.

## Supplementary Material
