# Correction: Surface enhanced Raman scattering for the multiplexed detection of pathogenic microorganisms: towards point-of-use applications

**DOI:** 10.1039/d1an90082j

**Published:** 2021-09-22

**Authors:** Matthew E. Berry, Hayleigh Kearns, Duncan Graham, Karen Faulds

**Affiliations:** Centre for Molecular Nanometrology, Department of Pure and Applied Chemistry, Technology and Innovation Centre, University of Strathclyde 99 George Street Glasgow G1 1RD UK karen.faulds@strath.ac.uk

## Abstract

Correction for ‘Surface enhanced Raman scattering for the multiplexed detection of pathogenic microorganisms: towards point-of-use applications’ by Matthew E. Berry *et al.*, *Analyst*, 2021, DOI: 10.1039/D1AN00865J.

The authors regret that an incorrect version of Table 1 was included in the original article. The correct version of Table 1 is presented below.

**Table 1:** A summary of the general approaches and studies discussed within this review

SERS approach	Summary	Performance
(1) Label-free detection	• SERS substrates for the capture of pathogens.	• Detection of three Gram negative bacterial strains (LOD 10^5^ CFU mL^−1^).^50^
	• Intrinsic vibrational fingerprint of pathogens detected (no Raman reporters used).	• Detection of *E*. *coli* and *S*. *aureus* in tap water and milk (LOD 10^3^ CFU mL^−1^, 10 min).^51^
		• Detection of multiple bacterial strains (LOD 1 CFU mL^−1^, 5 min).^52^
		• Detection of bacterial strains in mung bean sprouts (LOD 10^2^ CFU mL^−1^, 4 hours).^53^
		• Sampling and detection of bacterial pathogens from skin wound (LOD 10^6^ CFU mL^−1^, 8 hours (5 min for sampling)).^99^
		• Detection of meningitis pathogens in clinical CSF.^103^

(2) Microfluidics	• Sample preparation, reaction, separation and detection integrated into a single device.	• Label-free detection of *E*. *coli* and *S*. *aureus* in blood (LOD 10^5^ CFU mL^−1^, 100 CFU mL^−1^ in culture).^57^
	• Supports label-free and label-based detection.	• Label-free detection of three bacterial strains in serum (LOD 4 CFU mL^−1^, 15 min).^59^
		• Label-based detection of *S*. *enterica* and *N*. *lactamica* using DEP enrichment (LOD 70 CFU mL^−1^, 10 min).^61^
		• Label-based detection of three bacterial pathogens at millilitre scale in blood (LOD <100 CFU mL^−1^, 13 min).^63^
		• Label-based detection of *E*. *coli* using DEP enrichment (LOD 1 CFU mL^−1^).^65^
		• Detection of eight foodborne pathogens.^98^
		• Label-free detection of multiple viral strains in clinical nasopharyngeal swabs.^106^

(3) Nucleic acid-based detection	• Coupling of assays for the detection of pathogenic DNA with SERS substrates.	• Detection of meningitis pathogenic DNA (LOD in pM range).^72^ Detection of pathogen DNA in clinical CSF.^73^
	• SERS nanotags functionalised with DNA/RNA aptamers.	• Detection of KSHV and BA DNA using LFA (LOD in fM range).^74^
	• Supports label-based detection.	• Detection of *S*. *aureus* and *S*. *typhimurium* using aptamer-based magnetic sandwich assay (LOD 15 CFU mL^−1^).^78^
		• Detection of *E*. *coli* and *S*. *aureus* using aptamer-based magnetic sandwich assay in urine samples (LOD 50 CFU mL^−1^, 1.5 hours, 20 CFU mL^−1^ and 15 min for culture).^79^
		• Detection of *E*. *coli* using aptamers in beef samples (LOD 100 CFU mL^−1^, 20 min).^81^
		• Detection of influenzae A H1N1 in complex mixtures (LOD 97 pfu mL^−1^, 20 min).^82^
		• Detection of DNA from 11 common RTI pathogens using LFA (LOD in fM range, 20 min). Detection from throat swab.^110^
		• Detection of *S. enterica* and *L*. *monocytogenes* in milk, chicken and beef using LFA and RPA (LOD 22 CFU mL^−1^).^111^
		• Detection of plant pathogens on commercial crops using RPA outside of laboratory.^114^
		• Real time detection of MRSA genes using miniaturized PCR system.^115^

(4) Immunoassays	• Specific binding of pathogen antigen and antibodies coupled with SERS nanotags.	• Detection of Zika and dengue biomarkers using dipstick immunoassay (LOD 0.72 ng mL^−1^).^86^
	• Supports label-based detection.	• Detection of multiple viral strains using magnetic LFA (LOD 10 pfu mL^−1^).^89^ Detection in blood, serum and sputum (LOD 10^5^ pfu mL^−1^).
		• Detection of three bacterial pathogens using magnetic sandwich assay (LOD 10 CFU mL^−1^).^90^ Same LODs confirmed on portable system (1 hour).^108^
		• Detection of multiple viral and bacterial pathogens in serum using magnetic sandwich assay (LOD 10 pg mL^−1^).^91^

The Royal Society of Chemistry apologises for these errors and any consequent inconvenience to authors and readers.

## Supplementary Material

